# Speaking Truth to Power and Power to Truth: Reflections from the Pandemic

**DOI:** 10.1007/s11115-022-00606-z

**Published:** 2022-03-08

**Authors:** Flavia Donadelli, Robert Gregory

**Affiliations:** School of Government, Victoria University of Wellington, Wellington, New Zealand

**Keywords:** Science, Politics, Policymaking, Technocracy, New Zealand, Brazil

## Abstract

The complex relationship between science and politics has been a perennial issue in public administration. In this debate it is important to distinguish between ‘good’ and ‘bad’ politics, and between ‘good’ and ‘bad’ science. The Covid-19 pandemic has valorised the importance of science in shaping governmental responses, and has tended to contrast politics negatively with science. However, technocratic approaches to policymaking downplay the importance of politics in policymaking. Two case studies, of countries where there have been markedly different pandemic outcomes are used to illustrate the relationship between science and politics during this public health crisis – New Zealand and Brazil. In New Zealand there has been a positive and effective, if technocratic, relationship between science and politics, while in Brazil the relationship between the two domains has been fraught.

## Introduction

The tension between liberal democratic values, on the one hand, and governments’ need for scientific and technical expertise, on the other, has been a perennial theme in the scholarly literature of public administration (Waldo, 1948). It has reflected what Max Weber (1974: 139) saw as the historical process of ‘intellectualisation and rationalisation’, including the growing dominance of science and technology, meaning that ‘…one can, in principle, master all things by calculation’.

Whereas elected politicians are a defining feature of liberal democracy, the professionals and bureaucrats who are increasingly appointed to serve elected governments have come to acquire so much power and authority in their own right, that they have usurped or could usurp the legitimate role of elected officials. The Covid-19 pandemic has shown that effective governmental responses must rely heavily on expert scientific advice – especially that provided by epidemiologists, microbiologists, and immunologists.

In this article we do not attempt to pass any judgment on the validity or otherwise of scientific advice which addresses such central issues as community spread, infection, mortality, contact-tracing, and vaccination rates, or whether elimination, suppression, mitigation or other strategies are the most effective in containing the virus. Instead, we examine what can be seen as a ‘meta’ issue – the overarching relationship between science and politics in public policymaking, in regard to the pandemic, with comparative references to the cases of New Zealand and Brazil. New Zealand and Brazil have been chosen as two contrasting cases in the spectrum of pandemic management success and failure.

Examination of these two cases provides insights into the importance of ‘good politics’ for the successful use of ‘good science’ in policy-making and the role of ‘good science’ in checking ‘bad politics’. More specifically, we ask: to what extent can New Zealand’s generally successful performance in handling the pandemic be attributed to a mutually supportive and productive relationship between politics and science, in contrast to the Brazilian case, where this relationship and its outcomes have been markedly different?

After this introduction the first part of the article presents an overview of the theoretical debate around the politics - science divide, with a heuristic framework distinguishing among ‘good and ‘bad’ science and ‘good’ and ‘bad’ politics. The second part focuses on the politics of technocracy, with reference to the cases of New Zealand and Brazil in the management of Covid-19, and discusses the main implications of the preceding discussion for policy failure and success.

## PART ONE: Science and politics in public policymaking on Covid-19 – the good, the bad and the ugly

### Science, politics and rationality

In conceptualising his ‘four estates’ Price (1965) contrasted the scientific against the political, with the professional and administrative estates linking the scientific to the political. In his schema science is about the search for truth, while politics is about the exercise of power (where truth is often compromised); science values open debate and questioning, while politics may seek to close off debate; scientific research is conducted by highly trained experts but political action can readily be undertaken by ‘ordinary’ people with no specifically required expertise; science deals with the general, politics with the particular; science has to be principled, while politics tends to be opportunistic; and whereas scientists are tolerant of long-term inquiry politicians prefer to focus on the here and now. Moreover, scientists can become politicians much more easily than politicians can become scientists.

Similarly, science and policymaking can be seen as two distinct ‘communities’, separated by different languages, values and reward systems (Caplan, 1979; Amara et al., 2004; Newman et al., 2016.; Lofgren and Bickerton, 2021). According to this view several barriers of communication, priorities, time horizons and language would prevent the worlds of academic research and public policymaking from successfully interacting and learning from each other. In short, as with oil and water, science and politics do not easily mix.

So in cases like the current pandemic, effective policymaking will rest not only on politicians’ capacity for action but also on scientists’ ability to persuade politicians, and also the public at large. The skills of scientific analysis must combine with the skills of political advocacy, and both the demand and supply sides of this relationship should be prepared to interact in the first place (Nutley et al., 2009: 10–13; Donadelli, 2020). But the language of science is not readily communicable to lay citizens.

In this endeavour, much depends on the degree of scientific consensus, but political and social consensus on the relevance and urgency of the problem is also crucial. Where scientists develop a strong consensus about an issue that demands political and policy action and have been able to publicly convey their like-mindedness, politicians will find it more difficult to gainsay it. They can, however, still ignore the problem and keep it out of the political agenda. This is clearly apparent, for example, in the case of public policymaking on the effects and amelioration of climate change, where most politicians while professing to accept the science have nevertheless been slow to act (Ezrahi, 1980).

Unlike climate change (though perhaps less so in recent years), the main effects of the pandemic are obvious to all – infection, illness, hospitalisation and – far too often - death. So governments have been compelled to act, or be seen to be doing so. Some have acted decisively and immediately, others much less so. In doing so they have been able to draw upon a strong body of consensual advice proffered by epidemiologists, microbiologists and immunologists, among others. In most countries, contrary scientific advice has been marginalised in public debate, while in others, scientific controversy has been further fueled by political controversy, aggravating levels of uncertainty and stalling both decisive political action and social compliance with preventive measures. For example, scientific advocates of ‘herd immunity’ in countries like Sweden and Britain had their brief time in the political sun, while in New Zealand one university epidemiologist who was publicly critical of the government’s elimination strategy quickly became a professional outcast.^1^ In Brazil, on the other hand, political leaders such as president Jair Bolsonaro, a far right former military officer, have made a deliberate effort to emphasise scientific controversy, by publicly questioning the safety of vaccines or the effectiveness of masks^2^, and by directly opposing what other countries have been presenting as relatively consensual and ‘mainstream’ scientific knowledge.

Where high levels of scientific consensus do not exist policymakers can play off against one another expert advisers who promote different and often conflicting theories. At the time when concern over climate change was beginning to gain public attention, mainly in the 1980s, the scientific consensus was then more emergent, more contested, and less embraced by politicians driven by different partisan and electoral agendas. This has been even more true of political advice generated by the social sciences, including that discipline which has aspired to be the most like the natural sciences – economics. Economic policymaking is inescapably political in nature, with consensus being based as much on ideological preference as it is on scientific analysis, with political conflicts among key individuals, schools of thought, and vested interests (Carter, 2020; Parker, 2006). Ironically, the ideological basis of economists’ public choice theory, for example, which was widely invoked during the crusade to ‘reform’ governmental bureaucracies in the 1980s and 90s, to justify a ‘rolling back of the state’, has been discredited by the pandemic. Covid-19 has clearly shown that strong governmental capacity is essential in effectively responding for the public good.

Lindblom demonstrated in his seminal article, ‘The Science of Muddling Through’, that political rationality is less about means-ends choice, than it is about the quest for collective agreement on political and policy action (Lindblom, 1959). Those involved in policymaking do not have to agree on the ends, but if there is to be any action at all then they can agree to act, for their own different reasons. This is the art of coalition-building, and not the application of scientifically-generated and conclusive knowledge about the ‘one best way’ of achieving any particular end (Gregory, 1993).

The so-called ‘rational model’ of public policymaking is notable not for its promise of optimal outcomes, but for its limitations. Ideally, it can drive public policymaking in circumstances where four essential requirements are simultaneously met. There must be agreement on the goals to be achieved; there must be agreement on the means of achieving them; conclusive knowledge must be available about crucial causal relationships between the goals and the means of achieving them; and there must exist sufficient political desire to act. It is not hard to see, therefore, that only in rare or abnormal policymaking circumstances can these four conditions be present.

At first glance the current pandemic appears to offer a near perfect case of rational policymaking, similar perhaps to the programmes to eliminate or control the spread of other diseases, like poliomyelitis, tuberculosis and smallpox. Virtually everyone wants the spread of Covid-19 to be stopped or slowed down; but science is still learning while it advises, and governmental actions continue to be shaped by rapidly changing circumstances, which have been greatly complicated by political disputation over various lockdowns, and vaccination rates and priorities (Lewis, 2021). The goals also change. In New Zealand, as elsewhere, the original purpose in 2020 was to ‘flatten the curve’, that is, to slow down the infection rate. The government’s aim soon changed into ‘elimination’, that is, zero tolerance of any community outbreak, and by the later months of 2021 was beginning to shift back to ‘flattening the curve’ and then transitioning to suppression, based on the quest for high vaccination rates. In Brazil, president Bolsonaro initially dismissed Covid-19 as a ‘little flu’ and acted as a fierce opponent of any restrictive measures.^3^ He later changed his discourse and attitude to such things as mask-wearing and the purchase of vaccines, as a response to the successful public re-appearance of his main political opponent, Lula da Silva^4^.

The original virus has mutated to the more transmissible Delta and Omicron variants, and the vaccines’ effectiveness against all variants continues to be examined. So governmental actions to control the spread of the mutating virus appear similar not to a rational model or even a ‘boundedly’ rational one (Simon, 1990), but like Napoleon at the Battle of Borodino, as depicted by Tolstoy’s *War and Peace –* having to rely upon uncertain, ever changing, and often redundant information about the battle’s progress (Schön, 1971: 223). Scientific evidence, significantly contested, continues to proliferate, on all the critical issues – including viral mutations, the effectiveness of vaccines in preventing contraction, reducing transmission, and allaying the impact on people’s health if contraction occurs. As one Covid-19 expert has commented, ‘We’re kinda building the [Covid response] plane as it’s flying’.^5^.

Nor is this metaphorical ‘plane’ being developed in a political vacuum. In most countries where governments have taken steps to control if not seal the borders, including in New Zealand, they face strong political pressures to relax controls, implement ‘travel bubbles’ among different countries or jurisdictions, allow the return of citizens living overseas, and – in particular – to try to maintain acceptable levels of economic performance. Such political pressures are not easily assuaged by arguments that the best way to maintain economic productivity is for people to stay alive and well.

In all of this, ‘muddling through’ would seem to describe this reality at least as well if not better than the rational model, which demands much higher levels of conclusive knowledge and political commitment than are often available. On the other hand, effective control of community spread requires quick and decisive governmental action, which must be supported by high levels of willing social compliance of a kind that is rarely found in a liberal democratic polity. There must be high levels of political support both for the purposes of anti-Covid policy and for the means of attaining those ends. Such support may be less easy to sustain today than in earlier decades, when the pace of life was slower, people travelled much less, the internet, smart phones and social media had not become addictive, when there were far fewer sources of instant gratification, like fast food outlets and movie platforms, far fewer leisure-time options, and when more people enjoyed their leisure by reading books.

### Good and bad science

That said, the valorisation of science – often expressed as ‘keep [partisan] politics out of public health!’ – during the pandemic should be reconsidered. Of course, public health is profoundly political at any time, so if praise of science is accompanied by a reflexively reactive denigration of politics then society will pay a price, if people come to believe that science always provides an essential ingredient of ‘good governance’, and that politics is by contrast a dishonourable game in which the idea of the public interest is simply a fig leaf that disguises a self-regarding search for political power.

In contrasting science and politics, and in identifying the key values and norms of each domain, it is also possible to see how each can corrupt the other in their mutually constitutive relationship in public policymaking. Heuristically, there is good and bad science as well as good and bad politics. Without getting into Popperian epistemological debate about science and non-science, and concepts such as verification and falsification, and inductive and deductive research, it can be said that good science applies rigorous analytic methodologies, produces honest findings which can be equally honestly challenged by other such scientists, keeps open the possibility that results may be confounded by further research, and genuinely venerates an idea of truth (as philosophically loaded as it may be) as the lodestar of scientific activity.

Nevertheless, these key elements of science are idealistic, in the sense of a Weberian ‘ideal type’, for as Kuhn’s (1962) influential work argued, scientific progress is itself highly politicised. Scientists are jealous of their own reputations as good scientists, sometimes resistant to findings that run contrary to their own conclusions, and committed to maintaining an existing scientific ‘paradigm’ against the emergence of a new one that would supplant it. Scientific tribalism can be no less common than political tribalism. It just takes a different form. So rather than being completely antithetical, science and politics – as human activities – have more in common than is usually supposed. The role that scientists can have in politics, moreover, can range from ‘honest brokers’, when scientists effectively present all available data and expand politician’s scope of choice, to ‘policy advocates’, when scientists directly advocate for political decisions, thus restricting or biasing political choice (Pielke, 2007).

While it is more difficult to insulate social science research from political influences and easier to insulate natural science from immediate political considerations, nevertheless the latter can also be used to pursue political and ideological agendas. This is where power shapes ‘truth’, not where truth speaks to power. An obvious example is Nazi eugenics, a pseudoscience consigned to the same scientific dustbin that today contains phrenology, astrology, alchemy, various forms of alternative medicine, alongside theories promoted by the Soviet agronomist and biologist Trofim Lysenko, which were central to the USSR’s disastrous collectivist agricultural reforms in the 1930s.

In the natural sciences research can be and often is shaped by demands that are thrown up politically, the urgent need to control Covid-19 being an obvious case in point. People expect that their governments will bring to bear the resources of the state to cope with the pandemic, expectations which, as the world has seen, have not always been met, and not only in ‘failed states’.

### Good and bad politics

Liberal democracy provides the frame of reference for the good politics of what Popper (1945) called the ‘open society’, though it does not in and of itself preclude bad politics. Constitutional laws and conventions are the *sine qua non* of political institutions which enable and sustain impartiality, arguably the key value in liberal democratic governance (Rothstein and Torrell, 2008), and ‘the opposite of corruption’^6^. This is analogous to the impartiality of good scientific research. Freedom of association and freedom of speech are essential in political processes and are necessary but not sufficient conditions for what Habermas (1991, 1991a) calls ‘communicative rationality’, enabling citizens in a polity to seek consensus under non-repressive and non-suppressive conditions. His notion is more idealistic but not dissimilar to Lindblom’s idea of political rationality, mentioned above, and is to be found in no liberal democracy in pristine form. Assessed against Habermasian criteria some democracies can be judged to be, if not good or bad, then at least better or worse.

Good politics provides a supportive context in which good science can flourish, where the canons of good science are more likely to be respected, and where politicians will be less inclined to interfere in scientific research in order to shape findings that support their own ideological positions. Here the separation between Price’s scientific and political estates is satisfactorily maintained, so while there can be cross-fertilisation there is little cross-contamination between the two domains. This relationship is much less likely to be found in totalitarian or highly authoritarian political systems, where bad science is fostered by bad politics, with science being used for political purposes.

Good science can play a major role in challenging bad politics, where it has the political freedom to do so. The persistent message from climate science has gradually shifted popular understanding of the issue and the stakes involved, and political responses have become more positive. What Vickers (1983) called ‘the appreciative system’ in public policymaking has, in the case of climate change policy, been markedly changed over time by a political response driven by scientific insistence. It is an example of good science checking bad politics, bad in the sense that while liberal democratic conventions and norms have not necessarily been compromised, policymaking has been captured by strong vested interests, in a manner that obstructs the Habermasian vision.

As Kuhn showed, strong scientific consensus can consign collegial critics to the margins, a form of excommunication that is not too dissimilar to the closing down or exiling of political critics. After all, before Copernicus scientists believed that the Earth was the centre of the universe. Good politics therefore provides scientists languishing on the edges of respectability a platform from which orthodoxy, or Galbraithian ‘conventional wisdom’, can be challenged.^7^

**Fig. 1:**
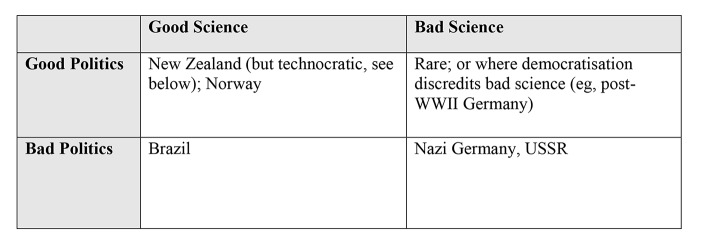
Good and bad science and politics in sovereign states

The ideal case in Fig. 1, where both good science and good politics prevail, is where optimal public policymaking is possible. The converse case is where bad science co-exists with bad politics, which is likely to be found in failed states or those which are profoundly anti-democratic, as in totalitarian states.

Bad science mixing with good politics is less likely to occur, because open political debate on scientific research and findings will expose quackery. And good science exists alongside bad politics. Good science is by no means precluded in such states, but when it is directed or strongly influenced by state politics it is less likely to be good science.

### Politics and Technocracy

‘Communicative rationality’ requires an inclusive common language, one that all members of a polity can understand and relate to. But scientisation and technocratisation give rise to exclusive languages, which enhance the power of those who can speak them against those who cannot. Scientific and quasi-scientific terminology becomes a part of common discourse – for example, genome sequencing – even though lay people do not understand the complexities embodied in the term. This may be no bad thing in the case of the pandemic, where the physical sciences are at work, but in social science technical terminology may often be politically disingenuous, especially when reified terms like ‘the economy’ or ‘financial markets’ obscure the realities of power relationships.

Technocracy is not so much a governmental structure as it is a state of mind. Some bureaucrats and politicians are more technocratically-oriented than others, meaning that they are more or less likely to believe in the efficacy of scientific and technical, theoretically-informed, means-end rationality (the ‘one best way’) in public policymaking. They embrace the language of problem-solving, even when so many policies can only aspire to cope with rather than to ‘solve’ social and other problems (Banfield, 1980).

Technocrats tend to eschew policymaking approaches that value open public debate and the quest for the collective agreement on action that Lindblom sees as the more democratically desirable alternative to the rational model (Fischer, 1990). They seek to insulate areas of policy from ‘political interference’, as in the case of central banks, for example (Marcussen, 2006; Gregory, 1998).

Most scientists see themselves as professionals, but while all professionals are not necessarily technocrats, they generally share a similar mindset, and tend to look upon political institutions and processes with disdain. This is not the cynical contempt for politics that is often expressed by lay people, though it may be, but derives from the professional’s belief in precision and calculation. In Mosher’s words, ‘Professionalism rests upon specialized knowledge, science, and rationality. There are *correct* ways of solving problems and doing things. Politics is seen as constituting negotiation, elections, votes, compromises – all carried out by subject-matter amateurs’ (Mosher, 1968: 109, emphasis in original).

Mosher was addressing the professionalisation of public bureaucracies, the populating of office by more and more government employees who can claim professional status, which is based primarily on several years of specialized study in a tertiary institution. Professionals have the formal qualifications to confirm their competence, and possess glossaries of acronyms which have over the past 20 years been proliferating at an exponential rate, especially during the pandemic. What can be called ‘acronymic language’, inaccessible to most lay people, is a threat to democratic processes.

Today’s bureaucracies are a far cry from those of Max Weber’s time, when technical expertise was exercise mainly by clerks, but professionalisation and scientisation are consistent with Weber’s description of ‘rationalisation’ – the progressive displacement of the substantive rationality of ends and purpose with the instrumental rationality of means – as (arguably) ‘the iron cage’ of modernity (Gregory, 2007). Against this background Covid-19 has impelled a more obvious shift to technocratic governance, because the need for scientific and technical authority in public policymaking is self-evident.

Technocrats involved in public policymaking – which is inherently and ineluctably political – are often obliged to speak as if they were quasi-scientists, in their claim to know what’s best. So the idea of ‘evidence-based policy’ has enjoyed much plausible appeal in recent years, in the face of overwhelming evidence, if anyone cared to look for it, that most political disputation is based primarily on beliefs rather than on conclusive evidence. The problem facing those few politicians who see themselves as dispassionate imbibers of hard evidence, untainted by the push and pull of partisan conflict, and dedicated to ‘the public interest’, is that they forever run the risk of being rigorously irrelevant. The politician *qua* scientist seeks to act on the ‘high, hard ground where [they] can make effective use of research-based theory and technique’ but in doing so become drawn back into the ‘swampy lowland where situations are confusing “messes” incapable of technical solution’ (Schön, 1983: 42).

The belief that ordinary people who inhabit the ‘swampy lowland’ cannot be trusted to understand complex issues has to be set aside the false claims of ‘experts’ who may hubristically and fallaciously claim access to certain knowledge. Technocratic overreach can have disastrous consequences, as numerous critics of technocracy and meritocracy have shown (Markovitz, 2019; Sandel, 2020; Halberstam, 1973; Shapley, 1993).

Moreover, because in liberal democracies politicians are under constant and usually irresistible pressure to act in the present rather than defer to future knowledge, they tend to focus their attention on present rather than longer-term circumstances (Boston, 2017). They cannot wait until scientific research throws up conclusive evidence, if it ever does, about the problems they have to deal with (though they may set up formal inquiries to avoid taking immediate action).

## PART TWO:

### The cases of New Zealand and Brazil

Why some countries have adopted a more technocratic approach than others in regard to the pandemic – meaning that technocratic authority has been more pronounced in some and much less so in others – would seem to depend on a range of political, institutional, social and constitutional factors, and on diverse political cultures. There is no space here to explore these differences, suffice it to say that whereas in New Zealand a strongly technocratic approach has been adopted and politically accepted, in Brazil this has not been the case, and the government’s response to the pandemic has been driven overwhelmingly by political considerations.

At the time of writing, according to *Worldometer*, New Zealand had a Covid-19 death rate of five deaths per million people, compared to Brazil’s 2,804 deaths per million. High levels of public compliance were generated in New Zealand in 2020, with the prime minister appealing regularly to ‘the team of five million’ to cooperate in ‘eliminating’ viral spread. However, after a major Delta outbreak in the highly populated and economically crucial Auckland region in August 2021 the government found itself under much more political pressure to loosen the restrictions it imposed over several weeks.

By contrast, in the Brazilian case there has never been political or scientific consensus in addressing Covid-19. Wildavsky (1979, Chap. 5) has argued that rationality in public policymaking is not to be understood as an instrumentally future-oriented and goal-driven selection of means and ends, but is as much a retrospective process of explaining and politically justifying actions which have emerged from a complex decision-making process. This is apparent in how the Brazilian government handled the discourse around vaccines. The president went from claiming in December 2020 that those taking the vaccine ‘would turn into alligators’^8^ and that he would not take the vaccine himself, to praising his own government, in March 2021, for its progress in vaccinating the population, as if this had been the plan all along.

In New Zealand, the government’s catch cry of ‘go hard, go early’ was emblematic of its strategy, which emerged as a mixture of informed responses to a variety of changing and uncertain circumstances. If the government could not be sure about what it was doing – after all, the pandemic was a completely new experience for all concerned – then it had to speak *as if* it did. The inevitable impact of a dynamic and highly fluid set of political preferences and priorities on the use of science cannot, therefore, be ignored. Despite being inherent to the nature of political action, bargaining and negotiation processes can be conducted in many different ways and can lead to quite different scenarios when it comes to the use of scientific advice.

Such advice has been lauded where governments have been relatively successful in responding to the virus, as in New Zealand, and it has also been praised *in absentia* where governments have failed to do so, as in Brazil. In the former case politicians were seen to have done a good job by drawing upon and applying sound scientific advice, while in the latter case politicians have been condemned for largely disregarding or directly opposing it. In the former a technocratic approach has prevailed for the common good, but in the latter politics has prevailed against the public good, as will be discussed below.

Figure 1 depicts the heuristic matrix of good and bad science and good and bad politics, in sovereign states.

In New Zealand the top health bureaucrat is the director-general of health (Dr Ashley Bloomfield, a medical professional) who heads the Ministry of Health. He has exercised his emergency powers under the Health Act 1956, and the Covid-19 Public Health Response Act 2020, allowing him to command medical officers of health in each of the country’s 20 District Health Boards (DHBs). So this has not been normal governance in normal times (Gregory, 2021).

The Labour Party-led cabinet in 2020 and its Labour Party successor in 2021 have been heavily reliant on Bloomfield’s expert advice, and that provided through him by other epidemiologists and statistical modellers. This reliance has been repeatedly confirmed publicly by the prime minister, Jacinda Ardern, who has prefaced her announcements with, ‘On the advice of the director-general…’, seemingly to say that ‘politics’ has rightfully been taken out of these science-based decisions. But this is hardly the case, because the government has to weigh a range of factors, including the economic effects of lockdowns. So its decisions are inescapably political, rather than purely scientific, choices.

This was confirmed publicly early in November 2021 when Ardern changed her phraseology to, ‘After a discussion with the director-general [of health]’ the government had decided to lower the alert level in Auckland. Questioning by journalists confirmed that Bloomfield had advised against doing so. The effects of political pressures on the government, including protests by many Aucklanders, had become more apparent. By this time the government had shifted its strategy from on of ‘elimination’, as applied during 2020, to ‘suppression’ or ‘containment’, given that the Delta outbreak had now made it impossible to keep the virus out of the community. Its urgent push for high vaccination rates, with the Pfizer vaccine, had now become the key plank of its new approach. The former alert level structure – with level four being full lockdown as had applied across the country in March-April 2020, was now replaced by a Covid-19 Protection Framework, colloquially known as the ‘traffic light’ system, whereby different regions had greater or lesser restrictions placed on them, largely based on factors such as vaccination, infection, and hospitalization rates. A ‘red’ area was most heavily constrained – by way of limits of gatherings, access to bars and cafes, retail outlets and so on being dependent on having an official vaccine confirming that the holder had received their double vaccination. Life in ‘green’ locations could proceed as in 2020, when there was no Covid-19 in the community, but by the end of 2021 no part of New Zealand was classified as ‘green’. ‘Orange’ regions were less constrained than ‘red’ ones and more so that ‘green’ ones would have been.

The relationship between the prime minister, the Covid-19 response minister, and Bloomfield is fully consistent with the conventions of responsible government embodied in New Zealand’s Westminster-styled parliamentary democracy, with its unitary governmental system. However, it is also a case of virtual technocratic governance, so unusually reliant is the political executive on scientific advice in this case of public policymaking. In this the New Zealand government has tried to strike a politically acceptable balance between the ‘precautionary principle’ and the ‘principle of necessity’. That is, it has decided to be ‘better safe than sorry’ while at the same not restricting people’s rights and liberties disproportionately to the risk of the virus overwhelming the country’s public health system and sharply increasing the mortality rate (Raposo, 2021). Empirical research confirms that the public policy choices made during the pandemic involve ‘morally problematic trade-offs’ (Belle and Cantarelli, 2022).

This political-technocratic approach to the crisis, as also in Norway (Christensen and Laegreid, 2020), has so far been politically rewarded. Although New Zealand’s 2020 general election was postponed for a month, from September to October, because of the pandemic, it resulted in a landslide win for the Labour Party, which now enjoys the first single-party Parliamentary majority since the introduction of proportional representation in 1996. Undoubtedly, this electoral success was firmly grounded on the Labour-led government’s performance in handling the pandemic, and the high profile leadership of a widely popular prime minister.

Although the effects of bad politics can be disastrous, the fact that good science can check bad politics over time is well exemplified by the Brazilian case. Brazil is a relatively new liberal democracy, re-established in 1985 after almost 20 years of military dictatorship. It has a federal political system, with 26 states and one federal district. In healthcare, the federal government retains responsibility for publishing guidance and information, buying essential resources, and providing technical assistance to states. Its role is essential in aiding states, especially in poorer areas of the country. Brazilian constitutional laws and conventions have, over the past three decades, enabled reasonable levels of political and civil rights, as well as freedom of speech and social debate.

This relative stability came under strain, however, after president Dilma Rousseff’s impeachment in April 2016, and the election of Bolsonaro in January 2019. These political events were followed by a new wave of political and civil rights conflicts. Atrocities such as the murdering of the Brazilian politician and human rights/LGBT activist Marielle Franco in 2018, and the murder of more and more indigenous rights leaders and environmental activists, show that the current Brazilian politics is far removed from the Habermasian ideal of an impartial and inclusive communicative democracy. 9,10,11.

Not surprisingly, when it comes to the impacts of ‘bad politics’ on the management of Covid-19, Brazil is one of the most representative world cases. Unlike New Zealand, where the public has been mostly compliant and supportive of the often draconian measures proposed by the government, the Brazilian population and local federal governments have been highly polarised and noncompliant. *Worldometer* had recorded a death toll of about 600,000 and about 21.5 million cases by October 2021, and the country had been through a series of medical system collapses and through several levels and types of social restrictions.

Brazil’s central government has consistently denied the seriousness of the Covid-19 pandemic. This denial led to a federal crisis, in which state governors published their own restriction guidelines, bought vaccines and other resources, and even published basic information after the federal ministry of health stopped publishing Covid-19 related data on its official website^12^. Scientific advice has been constantly delegitimised by Bolsonaro’s government (for example, regarding mask usage and the size of public gatherings^13^), but local initiatives such as the Northeast or the Sao Paulo scientific committees have contributed to the creation of local restriction protocols and the diffusion of sound information. On numerous occasions state governors had to judicially secure their decision-making autonomy in what became a highly confrontational and fragmented dispute between local and central governments.

Bolsonaro himself fostered scientific dissensus around the management of Covid-19. In May 2020 the minister of health, Nelson Teich, an oncologist, was forced to resign after opposing, on scientific grounds, Bolsonaro’s and the Federal Council of Medicine’s preference for the wider use of the anti-malarial drugs hydroxychloroquine and chloroquine as treatments for severe cases of Covid-19. Bolsonaro deliberately delayed the purchase of Covid-19 vaccines, and publicly questioned their safety and efficacy. By 2020 other Latin American countries such as Chile and Colombia had begun to negotiate the purchase of vaccines, but Brazil declined a 70 million dose contract offered by Pfizer^14^, causing a procurement delay that is now being investigated in a formal senate inquiry^15^.

Brazilian research institutes such as Fiocruz and Butantan started to negotiate their own independent agreements. Fiocruz, from Rio de Janeiro negotiating the purchase of the Oxford-AstraZeneca vaccine, and the Sao Paulo-based Butantan negotiating the purchase of the Chinese Sinovac and the technological transfer for local production of the Brazilian CoronaVac. The ministry of health approved Fiocruz’ purchase but vetoed Butantan’s negotiations with China, arguing that the Chinese vaccine was unsafe. After a heated political clash between Sao Paulo’s governor Joao Doria and Bolsonaro, CoronaVac was approved and became the first Covid-19 vaccine to be applied in the country, in January 2021. By October 2021, 79,65% of Sao Paulo’s state population had received the first dose, while only 50,85% of the people in poorer states such as Roraima had received one 16.

In short, Brazil is a clear case of the domination of scientific debate by political preferences, in the case of Covid-19, and stands in marked contrast to New Zealand’s example of technocratically-dominated politics. While both countries could invest in a more democratically healthy balance between science and politics, the pandemic experience shows that the public good is threatened much more by scientific negationism than by competent technocratic management.

### Relative policy success and failure: a moveable political feast

Policy success and failure can be measured in different ways, and in the case of Covid-19 circumstances are so fluid that any judgements about ‘success’ and ‘failure’ can only be tentative. The Bloomberg Covid Resilience Ranking (CRR) of 53 countries and jurisdictions uses 12 data indicators, including virus containment, the quality of health case, vaccination rates, mortality rates, and the easing of border restrictions. At July 2021 New Zealand held third position and Brazil was listed at 35. However, by late September 2021 New Zealand’s ranking had dropped from first to 38th place^17^, as the country grappled with a Delta outbreak, while Brazil had moved three places up to 32. (It has been argued that the drastic change in New Zealand’s ranking, despite the fact that it has very low infection and mortality rates when compared to most other countries, reflects the CRR’s bias against measures which adversely affect business interests.^18^)

Although, New Zealand’s Labour government has led substantially in the political polls throughout 2021, its support has been declining markedly since the 2020 election, which was seen by many as ‘the Covid election’, a one-off political phenomenon (see Levine, 2021). The Delta outbreak of August 2021 meant that the country’s largest city, Auckland, was locked down for more than three months. The government therefore has faced even more pressure from badly affected business interests, from having to manage high MIQ demand from New Zealanders wanting to return home from other countries, and from people wanting to travel to and from Australia and some Pacific islands. At the same time, the government’s urgent programme of national vaccination, though generally successful – 90% of the population were fully vaccinated by mid-December 2021 – has aroused a vocal minority against vaccine mandates and the loss of personal liberties. Also, in late December 2021 the government was found by the Waitangi Tribunal to have breached the Treaty of Waitangi for ‘political convenience’ by rejecting the advice of the Director-General of Health and the Ministry of Health to take particular steps to safeguard Māori against the spread of the virus.^19^ Moreover, many people have complained that the shift from the alert level system of elimination to the ‘traffic light’ strategy of suppression has been confusing, despite the government’s concerted pains to explain the change and the reasons for it. In short, the politics of the pandemic had by the end of 2021 become a rapidly moveable feast.

Many factors contribute to the relative success or failure of different countries’ handling of the pandemic. Geography is one such factor, while others may be cultural and social and (healthcare) institutional, apart from being political. The respective roles of the news media and social media are also crucial. It may also be pertinent that while New Zealand is ranked first equal (with Denmark) in the Transparency International’s Corruption Perceptions Index of 2020, Brazil is in equal 94th place. Similarly, the rate of trust in government in Brazil, 36%, is much lower than the New Zealand rate of 63% (OECD, 2021). As Christensen et al. (2016) argue, effective crisis management demands high levels of governance capacity and public legitimacy.

There is no space here to elaborate on the relevance of these variables, except to say that geography is clearly a major difference, one that certainly affects the efficacy of Covid-19 responses. New Zealand is a small country of 5.2 million people, comprising three main islands, so its borders can be sealed quite quickly and effectively. It has extensive and relatively unpopulated rural spaces outside of its cities and towns. Brazil on the other hand with a population of 213 million is a huge land mass, and shares borders with 10 other countries. Cultural and social factors must also influence the different policy outcomes.

Nevertheless, it is inconceivable that any country could be relatively successful in controlling the pandemic within its borders without heavy reliance on epidemiological and immunological expertise, whether provided endogenously or exogenously. What may matter even more is the extent to which policymakers act on strongly consensual or highly conflictual scientific advice, and whether political leadership explicitly embraces scientific advice, as in New Zealand, or whether it undermines social trust in scientific advice, as in Brazil.

Hypothetically speaking, when policymakers are confronted by strongly conflicted scientific advice political considerations are more likely to influence their decisions than when the converse is the case. But where political polarisation is so extreme that it undermines trust in scientific advice, a moderate level of scientific consensus is not enough in itself to foster a productive relationship between science and politics.

## Conclusions

The Covid-19 pandemic has had disastrous consequences across the world. However, it may also have provided an opportunity to reflect on an ideal relationship between science and politics in public policymaking. The benefits of good science in dealing with the pandemic are self-evident, and to be highly valued, but so too are the advantages of good politics, especially in restraining technocratic and political elites from abusing their power. In immediately critical situations like the pandemic political power is usually placed in the hands of experts more than is normally the case in liberal democracies, and science is seen as a more desirable component of policymaking than is (mere) politics, but in the end politics is determinative, one way or the other. So, the task is to ensure that the advantages of good politics in public policymaking can prevail over misguided, and often ideologically-disguised, advice proffered by bad science. Similarly, if minimal democratic conditions exist, good science can check bad politics and prevent potentially disastrous outcomes.

## Footnotes

1
https://www.stuff.co.nz/national/health/coronavirus/125035835/the-scientist-and-the-rabbit-hole-how-epidemiologist-simon-thornley-became-an-outcast-of-his-profession?cid=app-iPhone.

2
https://www.ctvnews.ca/health/coronavirus/brazil-s-bolsonaro-warns-virus-vaccine-can-turn-people-into-crocodiles-1.5237678.

3
https://edition.cnn.com/2020/05/23/americas/brazil-coronavirus-hospitals-intl/index.html.

4
https://time.com/5946401/brazil-covid-19-vaccines-bolsonaro/.

5
https://wamu.org/story/20/03/20/were-building-the-plane-as-its-flying-a-d-c-public-health-expert-on-the-coronavirus-response/.

6
https://ecpr.eu/Events/Event/PaperDetails/25020.

7 Albert Hirschman’s (1970) influential book on the moral choices of ‘exit’, ‘voice’ and ‘loyalty’ could be applied to scientific advisers who may feel that while they have the ear of policymakers their advice is being ignored, or worse, distorted for public consumption. It may be asked, for example, whether in 2019–2020 Drs Anthony Fauci and Deborah Birx should have resigned (exited) from president Trump’s White House taskforce on the pandemic and spoken up publicly (voice) about their dissatisfaction with the president’s pronouncements and approach, rather than seeming to stay ‘loyal’ to Trump by remaining in the group.

8
https://www.nzherald.co.nz/world/covid-19-coronavirus-brazils-jair-bolsonaros-bizarre-pfizer-vaccine-rant/2N6RNZUVUGGUWC42IHQQFE7NPM/.

9
https://www.theguardian.com/world/2018/mar/18/marielle-franco-brazil-favelas-mourn-death-champion.

10
https://news.mongabay.com/2019/12/murders-of-indigenous-leaders-in-brazil-amazon-hit-highest-level-in-two-decades/.

11
https://www.theguardian.com/environment/2020/jul/29/record-212-land-and-environment-activists-killed-last-year.

12
https://www.theguardian.com/world/2020/jun/07/brazil-stops-releasing-covid-19-death-toll-and-wipes-data-from-official-site.

13
https://www.nature.com/articles/d41586-021-01031-w.

14
https://www.brasildefato.com.br/2021/06/08/bolsonaro-refused-to-buy-pfizer-s-vaccine-at-half-the-price-paid-by-other-countries.

15
https://www.aljazeera.com/news/2021/5/24/squabbles-and-accusations-inside-brazil-covid-senate-inquiry.

16
https://especiais.g1.globo.com/bemestar/vacina/2021/mapa-brasil-vacina-covid/.

17
https://www.stuff.co.nz/travel/news/300418832/covid19-new-zealand-falls-37-spots-on-global-ranking-of-best-places-to-be-amid-the-pandemic.

18
https://www.newsroom.co.nz/comparing-covid-how-new-zealand-stacks-up.

19 The Waitangi Tribunal is a statutory body that investigates post-1975 breaches by governmental authorities of the Treaty of Waitangi, which was signed between many Māori tribes and the colonial government in 1840.
